# Topics of Public Interest

**Published:** 1911-09

**Authors:** 


### TOPICS OF PUBLIC INTEREST

---

### Dust Deals in Death

**Consumption Spreads in Dusty Trades. Government Making Study—Open Windows, Ventilation, and Cleanliness the Cure.**

A WARNING against the dangers of dust was issued in a statement made today by the National Association for the Study and Prevention of Tuberculosis, in which it is shown that the percentage of deaths caused by tuberculosis in dusty trades is more than double that for all employed men in the registration area of the United States.

As a result of the dangers from consumption to those exposed to various forms of dust, and at the request of the National Association, the United States Government has recently appointed a commission to work in coöperation with state authorities in making an investigation into the conditions of the metal mining industries in the United States, with special reference to diseases of the lungs. The work of the commission engaged in this special task will follow lines somewhat similar to those worked out by the Royal Commission of Australia, whose report was recently received in this country.

"Dusts are of three kinds," says the National Association; "factory, street, and house dusts." The statement refers to the results obtained through investigations made for the Bureau of Labor, by Frederick L. Hoffman. While among males generally in the registration area of the United States 14.5 per cent. of all deaths are from consumption, the mortality among grinders from this disease is 49.2 per cent., and in hardly any of the dusty trades is it below 25 per cent. The percentage of deaths from tuberculosis among all those exposed to metallic dust is 36.9 per cent.; to mineral dust, 28.6 per cent.; to vegetable fibre dust, 28.8 per cent.; to mixed animal and other forms of dust, 32.1 per cent.; to street dust, 25.5 per cent.; and to organic, or dust coming from the articles being manufactured, 23 per cent.

The statement speaks also of the dangers from house dust, especially in rooms that are not well ventilated. The Association warns against dry sweeping, and against the use of the feather duster, or other devices that scatter, but do not take up the dust.

Since the ordinary dust blown about in the streets is impregnated with disease germs, the National Association urges the adoption of methods that will prevent the further dissemination of such bacilli. It also urges for the coming months of fall and winter, more open windows and more fresh air in house, shop, and schoolroom.

---

### Insane Hospitals Lack Tuberculosis Accommodations

**Deaths from Consumption Double Ordinary Rate—Only One-Third Provide for Diseased.**

Out of more than 225 public hospitals for the insane, with a population of fully 150,000, only 70, or less than one-third of them, make any provision for their tuberculous inmates, and this too, when the percentage of deaths from this disease is very high. This is the substance of a statement made by the National Association for Study and Prevention of Tuberculosis.

Seventy hospitals in twenty-eight states, providing all told about 3,350 beds for tuberculous insane patients, sums up the provision made for this class of sufferers.

"When it is considered," the National Association says, "that the percentage of deaths from tuberculosis among the insane is from 50 to 200 per cent. higher than among the general population, according to the institution, the need for special provision is apparent." Autopsies made in New York State Hospitals and elsewhere show that tuberculosis is an active disease in about 20 per cent. of the cases, as compared with about one-half that percentage in a normal population. Superintendents of various hospitals in all parts of the United States testify that among the insane in institutions, tuberculosis is manifest in from 20 to 38 cases in every thousand. In the country as a whole about 10 to 15 people per 1,000 are afflicted with the disease.

Because of the habits of the insane, and the difficulty in teaching many of them the rules of cleanliness, the National Association says that separate buildings for the tuberculous should be provided in every hospital for the insane where tuberculosis is at all prevalent. In many cases, where insane persons through outdoor sanatorium life have been cured of tuberculosis, their minds have also been helped, and some have been discharged as mentally sound, who would otherwise have died, both insane and tuberculous.

---

A New Type of Ambulance.—The Ernest Wende Hospital for Contagious Diseases is installing two automobile ambulances. It is altogether likely that they will inaugurate a general advance in prophylaxis. The exterior of the ambulance is merely a shell to contain the receptacle for the patient. This consists of aluminum and is very light, and for further convenience in handling, is mounted on roller-bearing wheels. Five such receptacles are provided, one for each of the common contagious diseases, and one for miscellaneous use. These receptacles are kept in a steel sterilising chamber, of as many compartments, except when actually needed.

This ingenious invention for minimising the risk of spreading infection during the transportation of patients originates with Dr. Walter S. Goodale, the Superintendent. Buffalo may well be proud of the innovation, especially as Dr. Goodale is too modest to show such a weakness.

---

The Treatment of Pulmonary Tuberculosis Based on the Assumption that the Dietetic Cause of the Disease is Lime Starvation.—John F. Russell, New York, presents the results of the dispensary treatment of working people at work. Patients to be admitted must have uncomplicated pulmonary tuberculosis, with a temperature below 100° in the evenings. No patients are received who have ulcerative laryngeal tuberculosis, or whose sputum does not show tubercle bacilli. These germs are considered to have disappeared if after six examinations none is found. A table is given showing the results of treatment in twenty patients, most of whom now show no physical signs. Of twenty-one other cases no report is made. Fifty-five per cent. of the cases are cured. This is a larger percentage than in most sanatoria. The highest percentage of cases reported cured in a sanatorium is 34. It is far better to spend money in curing wage earners while at their work than to establish sanatoria for the incurable.—*Medical Record,* July 1, 1911.

